# Energy disregulation, adropin as biomarker related with depressive disorder

**DOI:** 10.1192/j.eurpsy.2025.1336

**Published:** 2025-08-26

**Authors:** D. Krnic, S. Kojundzic, R. Roje, D. Krnic

**Affiliations:** 1 Psychiatry; 2 Radiology; 3 Surgery, UHC Split, Split, Croatia

## Abstract

**Introduction:**

Adropin is a unique hormone or biomarker that is produced in the liver, brain, and peripheral tissues such as the heart and gastrointestinal tract. It was discovered in 2008 by Kumar and others. It is encoded by a gene associated with energy homeostasis. Adropin plays a key role in the development of various CNS disorders such as stroke, schizophrenia, bipolar disorder, Alzheimer’s disease, and Parkinson’s disease.

**Objectives:**

Depressive disorder affects a large number of people worldwide, with symptoms such as loss of energy and motivation, sadness, fatigue, and weakness significantly impacting quality of life and functionality. Numerous studies have shown that depression has various neurobiological mechanisms. Deregulation of energy metabolism in the brain is one of the mechanisms associated with the onset of depressive disorder. Adropin can activate BDNF, and since BDNF plays a crucial role in neurogenesis, synapse formation, plasticity, learning, memory, and has a neuroprotective function, adropin may play an important role in the development of depression.

**Methods:**

We conducted a study in which we examined adropin concentrations in depressive disorder in individuals who were diagnosed with depression for the first time and compared adropin levels with healthy controls. We followed up with depressive patients after six months of introducing SSRIs (Selective Serotonin Reuptake Inhibitors) antidepressants.

**Results:**

The adropin values after the introduction of antidepressants and improvement of depressive symptoms were significantly higher after six months compared to the initial blood draw.

**Conclusions:**

Our study measured serum adropin levels in patients who had not previously been treated for depressive disorder. After introducing SSRIs (Selective Serotonin Reuptake Inhibitors) and six months after the initiation of therapy, we repeated the adropin measurements. The results showed a statistically significant difference between the first and second measurements. In the second measurement, adropin levels were higher than in the first. We can conclude that with the improvement of depressive symptoms, the serum adropin levels, as a neuroprotective biomarker, also increased.

**Disclosure of Interest:**

None Declared

